# Maxillary sinuses microbiology from patients with chronic rhinosinusitis

**DOI:** 10.1590/S1808-86942010000500002

**Published:** 2015-10-22

**Authors:** Karina Mantovani, Andréia Alessandra Bisanha, Ricardo Cassiano Demarco, Edwin Tamashiro, Roberto Martinez, Wilma Terezinha Anselmo-Lima

**Affiliations:** 1 MD. Graduate student (MSc). Faculdade de Medicina de Ribeirão Preto da Universidade de São Paulo; 2 MD. Resident physician. Faculdade de Medicina de Ribeirão Preto da Universidade de São Paulo; 3 MSc. Assistant physician. Faculdade de Medicina de Ribeirão Preto da Universidade de São Paulo; 4 MD. Graduate Student (PhD). Faculdade de Medicina de Ribeirão Preto da Universidade de São Paulo; 5 Associate Professor. Faculdade de Medicina de Ribeirão Preto da Universidade de São Paulo; 6 Associate Professor. Faculdade de Medicina de Ribeirão Preto da Universidade de São Paulo

**Keywords:** microbiology, sinusitis.

## Abstract

**Abstract:**

There isn't definitive and consistent data concerning the distribution of bacterial species in patients with Chronic Sinusitis (CS). The variability of the results from studies in CS may be due to the different techniques used as collection method, variations in culture methods, previous antibiotic use, and difficulty in distinguishing bacterial flora from pathogenic agents.

**Study design:**

Clinical prospective.

**Aim:**

To identify the incidence of microorganisms in patients with CRS by growing bacteria from the secretion of the maxillary sinus.

**Patients and Methods:**

Cross-sectional study in 62 patients that had undergone FESS for treatment of chronic sinusitis; cultures from the maxillary sinus were obtained.

**Results:**

62 samples, 33 (53.2%) had no growth; 29 (45.2%) counts of aerobic bacteria; one case (1.6%) of fungus growth; we did not find anaerobic bacteria. Pseudomonas aeruginosa was the one more frequently found - 8 samples (27.6%), Staphylococcus aureus and Staphylococcus epidermidis in 4 samples each; Streptococcus pneumoniae in 3 samples (10.4%); other Gram negative agents in 17 samples (31%).

**Conclusion:**

In the present study we concluded that Pseudomonas aeruginosa, other Gram negatives bacteria and Staphylococcus spp were the representatives of the bacterial flora found in the paranasal sinuses of patients with CS.

## INTRODUCTION

Despite the different studies approaching the subject of Chronic Rhinosinusitis (CRS), we still do not have a clear understanding of the true pathogenic mechanisms and agents involved in this disease. One of the investigation fronts has turned its attention to try to understand the inflammatory mediators involved in CRS^1^. Despite major progresses in this field, it is still not clear which is the ultimate agent responsible for triggering the upregulation of the eosinophilic and lymphocytic activities, which happens in CRS, which in its turn triggers the subsequent inflammatory events on the nasosinusal mucosa.

In an attempt to justify which would be the triggering factors of the inflammatory events, one of the current hypothesis blames infectious agents, especially bacteria and fungii, among the main agents responsible for the genesis and maintenance of CRS. Contrary to microbiology tests done in patients with acute rhinosinusitis, there is no definitive and consistent data on the real distribution of bacteria in patients with CRS. Result variability from CRS studies are due to the different techniques used as harvesting method, variations in culture methods, prior use of antibiotics and, especially, difficulties in distinguishing which are the colonizing agents and which are truly pathogenic, making it impossible to reach a definitive result today.

Because of the aforementioned reasons, we decided to study the incidence of microorganisms present in patients with CRS in our region, by means of a culture of maxillary sinus secretion, harvested with the use of an endoscope.

## PATIENTS AND METHODS

We carried out a cross-sectional study involving 62 patients, 30 men and 32 women, ranging in age between 13 and 78 years (mean of 45 years), diagnosed with CRS, seen at the ENT Ward from March of 2005 to September of 2006.

We included patients with CRS, diagnosed according to the European Consensus^2^, who did not get better after exhaustive clinical treatment (anti-histaminic agents; antibiotics; nasal flushing with saline, topical and systemic steroids), and those who were referred to functional endoscopic sinus surgery. We excluded those patients who had used antibiotic agents in the thirty days prior to sample collection, and those who had some anatomical change which would prevent us from seeing the middle meatus.

After anesthetizing the patient, the nasal cavity was thoroughly flushed with saline solution; cotton pads soaked in a 1:10000 vasoconstriction solution were then introduced in the nasal cavity and left there for ten minutes. Following that, we penetrated the maxillary sinus and collected secretion by means of a catheter connected to a syringe, which was introduced all the way to the sinus for aspiration purposes. The material harvested was processed following microbiological methods aiming at isolating anaerobic and aerobic bacteria and fungi.

The material used to find the aerobic bacterial was sowed in Agar Blood medium (Müeller Hinton Agar + 5% of goat blood) Mac Conkey (Müeller Hinton Agar, peptone, billiary salts, purple crystal, lactose and neutral red pH) and Ni (simple Agar and 7.5% of NaCl), incubated at 37°C, during 24 hours. For the identification of the microorganisms isolated we did tests in the VITEK® automated system, completing it with tests, when necessary, to characterize gender and species.

The material referred to study anaerobic bacteria at the time of harvesting was already introduced in an anaerobic blood culture flask and it was then transported to the lab. The flask was incubated in the BACTEC® device during seven days. If the device detected positive growth, we would run sub-culturing of the bacteria in Brussels Blood Agar added by L-cystine supplements in anaerobiosis at 35°C during 48 hours. When we confirmed the presence of strict anaerobe agents, the material was taken to the VITEK® device, which would then identify the gender and the species of the bacteria.

The material used to test for fungus was sowed in Sabouraud Dextrose Agar (SDA) with the adding of chloramphenicol and in Mycosel®. SDA was incubated at 37°C during 30 days, while Mycosel remained for 30 days in room temperature. They were read daily in order to check for fungus growth.

When we noticed fungus growth, it was immediately identified by means of its morphology and tests of assimilation and fermentation.

This study was approved by the Ethics Committee of our institution, in accordance with Process # 1930/97.

## RESULTS

**Figure 1:**
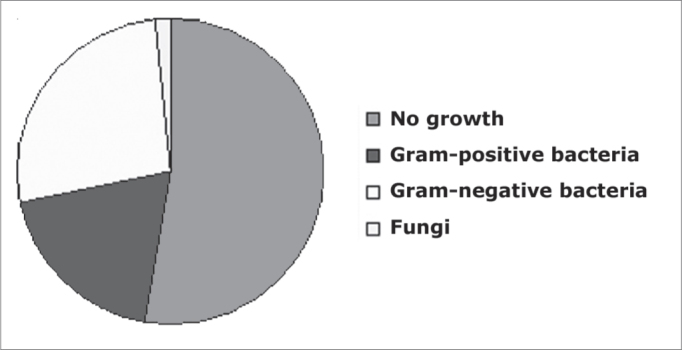
Distribution of the groups of microorganisms isolated from maxillary sinus secretion cultures.

Of the 62 samples studied, 33 (53.2%) showed no growth of microorganisms; in 29 (45.2%) there were aerobic bacteria; in only one case there was fungus growth; we did not find anaerobic microorganisms (Fig. 1).

Of the aerobic microorganisms found in the 29 patients, *Pseudomonas aeruginosa* was the most commonly found bacteria - in eight samples (27.6%). *Staphylococcus aureus* and *Staphylococcus epidermidis* were found in four samples each (13.9%). Streptococcus pneumoniae was found in three samples (10.4%), and *Proteus mirabilis* in two samples (6.9%). *Klebsiella pneumoniae, Escherichia coli, Streptococcus viridans, Enterobacter cloacae, Enterobacter aerogenes, Haemophilus* sp, *Haemophilus influenzae* and one unidentified Gram negative rod were found in each sample (3.4%). *Cryptococcus neoformans* was found in only one sample (Table 1).

**Table 1:** Aerobic bacteria found in 29 patients with positive culture.

Bacteria	Number of patients (%)
Gram-positive	
*Staphylococcus aureus*	4 (13,9%)
*Staphylococcus epidermidis*	4 (13,9%)
*Streptococcus pneumoniae*	3 (10,4%)
*Streptococcus viridans*	1 (3,4%)
Gram-negative	
*Pseudomonas aeruginosa*	8 (27,6%)
*Proteus mirabilis*	2 (6,9%)
*Klebsiella pneumoniae*	1 (3,4%)
*Enterobacter cloacae*	1 (3,4%)
*Enterobacter aerogenes*	1 (3,4%)
*Escherichia coli*	1 (3,4%)
Gram-negative rod	1 (3,4%)
*Haemophilus sp*	1 (3,4%)
*Haemophilus influenzae*	1 (3,4%)

## DISCUSSION

In recent years, many studies published in the literature attempted to validate the culture done in samples harvested through endoscopy of the middle meatus^3^, ^4^, ^5^, ^6^, ^7^, ^8^, ^9^, ^10^, ^11^, and Jiang et al. (1993)^12^ studied and established the aspiration of the middle meatus.

The study carried out by Ozcan et al. (2002)^13^ showed a high correlation between the results from cultures made out of secretion harvested from the middle meatus and ethmoid/maxillary sinuses, and the former must be used in the routine investigation and monitoring of patients with CRS, in order to minimize treatment failure, thus increasing the effectiveness of antibiotic use.

Araújo et al.^10^ showed that in 80% of the samples harvested, both punctioning the maxillary sinus as well as aspirating the middle meatus, there was growth of the same microorganism. According to the same authors, these studies suggest that the culture after endoscopic harvesting of secretion from the middle meatus is a feasible alternative to anthral punction, for being effective in the identification of the pathogens and for being a non-invasive method in the etiological diagnosis of CRS.

Moreover, Jiang et al. (2002)^14^ stated that because the middle meatus drains the anterior ethmoid, frontal and maxillary sinuses, the bacteriology of this area better reflects the microbiology of the paranasal sinuses when compared to material from the maxillary punction.

The present study found a larger number of positive cultures for Gram negative bacteria (58.6%), and the most frequently found agent was *Pseudomonas aeruginosa* (28.6% of the positive cultures). We obtained 12 cultures with Gram-positive bacteria growth, when the most frequently found agents were: *Staphylococcus aureus* and *Staphylococcus epidermidis* (13.9% each).

This data found are not surprising and are similar to those of prior studies^15^,^16^, which showed that the most frequent agents in CRS were *Pseudomonas aeruginosa* and *Staphylococcus aureus*, besides other Gram-negative; and it also detected a higher frequency of *Pseudomonas aeruginosa* in patients with a past of function endoscopic sinus surgery.

Nonetheless, Nigro et al.^17^ found a predominance of coagulase-negative *Staphylococcus* (12.1%) and *Staphylococcus aureus* in 9.7%, probably due to differences in the collection or sowing methods.

*Staphylococcus epidermidis* is a known colonizer of the nasal cavities and Ozcan et al.^13^ advocates not including it in CRS bacteriology -it should be considered as contaminant.

*Enterobacteriacea* are considered CRS infection agents, although with a secondary role. *Streptococcus* viridans do not play any important role in CRS; however they can become pathogenic under opportunistic situations, since they produce b-lactamase, causing bacterial resistance.

In the present study, anaerobic agents were not found in any of the samples collected. Some studies^13^,^14^ also did not detect anaerobic bacterial growth; others state that the anaerobic bacteria are major contributors to the CRS disease process^15^, and, some showed the detection of anaerobes varying between 0% and 88%^17^.

One possible explanation would be that the hemoculture dishes used for primary detection were not adequate for this type of material (paranasal sinus secretion), eventually, because of excessive material dilution or for having some air being injected together with the secretion).

The ratio of negative cultures (53.2%) was a bit higher than the value found in previous studies, of about 40%^16^; however, some authors reported that the rate of bacterial growth in cultures may vary between 17 and 60%^18^. These differences may be due to the use of different transportation and sowing methods.

In our study, fungi represented only 1.6% of the cases, similarly to what was reported by Nigro et al.^17^; meanwhile, Araújo et al.^19^ reported 14%. Cryptococcus is an opportunistic yeast which is occasionally isolated from the paranasal sinuses secretion from AIDS and other immunosupressed patients.

## CONCLUSION

In the present investigation we can conclude that *Pseudomonas aeruginosa*, *Staphylococcus aureus* and other Gram-negative bacteria represent the main microbiota present in the paranasal sinuses of patients with CRS in our region.
